# Correlation of DTI tractography with electroanatomic mapping in normal and infarcted myocardium

**DOI:** 10.1186/1532-429X-16-S1-O68

**Published:** 2014-01-16

**Authors:** Choukri Mekkaoui, Marcel P Jackowski, Aravinda Thiagalingam, William J Kostis, Sonia Nielles-Vallespin, David Firmin, Himanshu Bhat, Jeremy N Ruskin, Timothy G Reese, David E Sosnovik

**Affiliations:** 1 Radiology, Harvard Medical School - Massachussets General Hospital - Martinos Center for Biomedical Imaging, Charlestown, Massachusetts, USA; 2 Department of Computer Science, University of São Paulo, São Paulo, São Paulo, Brazil; 3 Cardiology, Harvard Medical School - Massachussets General Hospital, Boston, Massachusetts, USA; 4 CMR Unit, Royal Brompton Hospital - Imperial College, London, UK; 5 Siemens Medical Solutions USA Inc., Charlestown, Massachusetts, USA; 6 National Heart, Lung and Blood Institute, National Institutes of Health, Bethesda, Maryland, USA

## Background

The tractographic propagation angle (PA) is a topographic measure of fiber architecture in the myocardium. We have previously shown in infarcted mice that a PA > 4° can be used to differentiate normal and infarcted myocardium [1] . Here, we extend these preliminary observations by 1) characterizing PA values in normal sheep and human hearts, and 2) correlating changes in PA with changes in myocardial voltage on electroanatomical maps of infarcted sheep hearts.

## Methods

Large anteroseptal infarcts were created in sheep (n = 5) by balloon occlusion of the left anterior descending coronary artery. Bipolar voltage mapping of the left ventricle was performed at 3 months using the CARTO^® ^3D electroanatomic mapping system. The hearts were then perfusion-fixed and excised. Cardiac diffusion tensor MR imaging (DTI) was performed on a clinical 3T scanner, as previously described [1] , with a resolution of 2 × 2 × 2 mm^3^, b-value of 2000 s/mm^2^, and 6 diffusion-encoding directions. This sequence was also used to image 5 normal sheep hearts and 5 *ex vivo *human hearts. Moreover, *in vivo *DTI in 11 human volunteers was performed using a stimulated echo single shot EPI sequence, as previously described [2] . PA values for each heart were computed along myofiber tracts using an adaptive 5^th ^order Runge-Kutta method. Since PA in the sheep hearts was more densely sampled than voltage, each voltage point was associated with a distribution of PA values.

## Results

**Figure 1:**
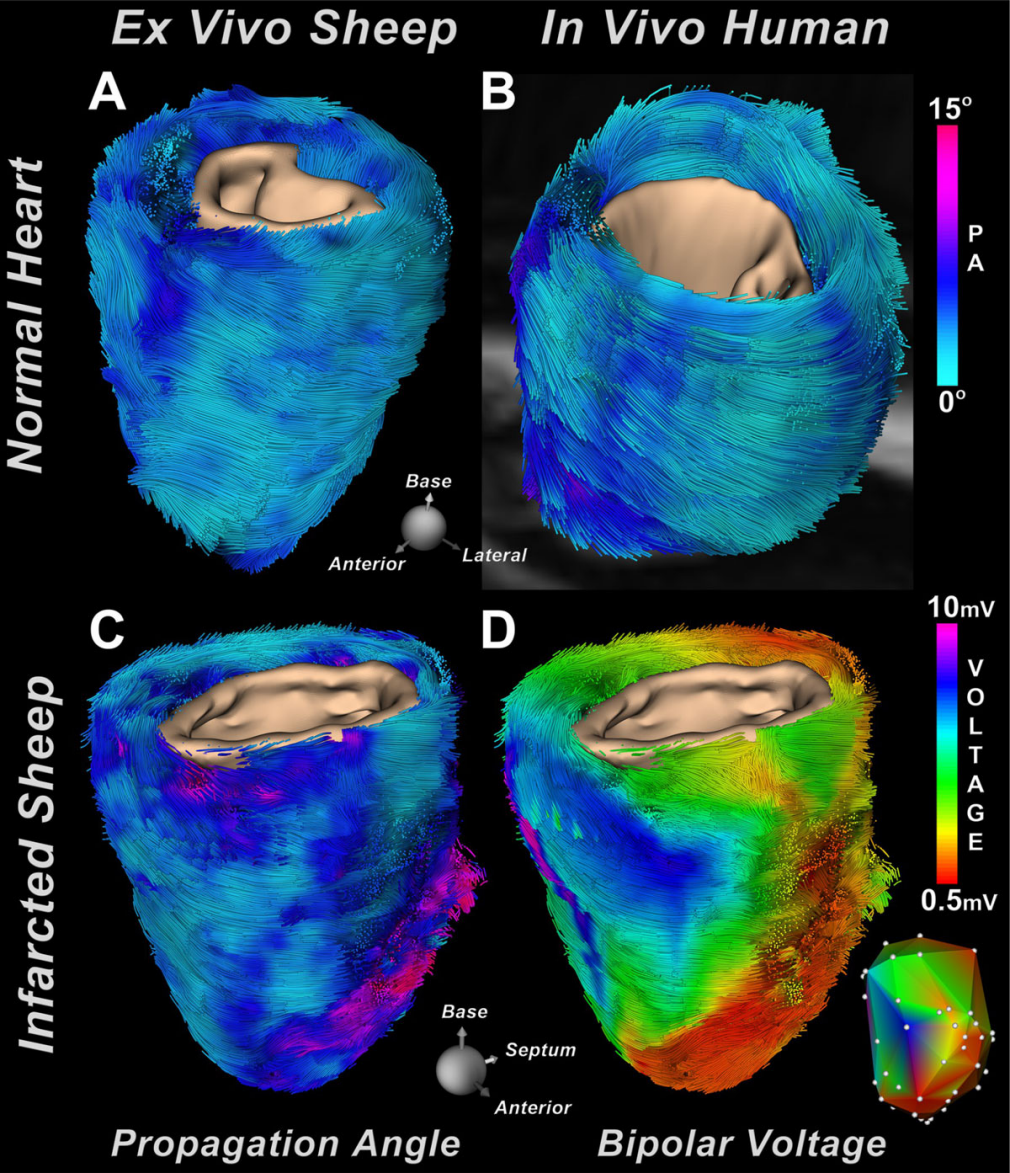
**Whole hearts show that PA values in **(A) **a normal sheep heart *ex vivo *are similar to those seen in **(B) **a normal human heart *in vivo *during systole**. **(C) **Tracts of an infarcted sheep heart color-coded by PA show that PA is significantly increased in the infarct zone. **(D) **Tractograms color-coded by bipolar voltage show that regions of low voltage correlate well with regions of high PA. The microstructural and electrical transitions between normal myocardium, border zone and infarct can be clearly seen. The inset displays the original bipolar voltage map and measurement locations.

**Figure 2:**
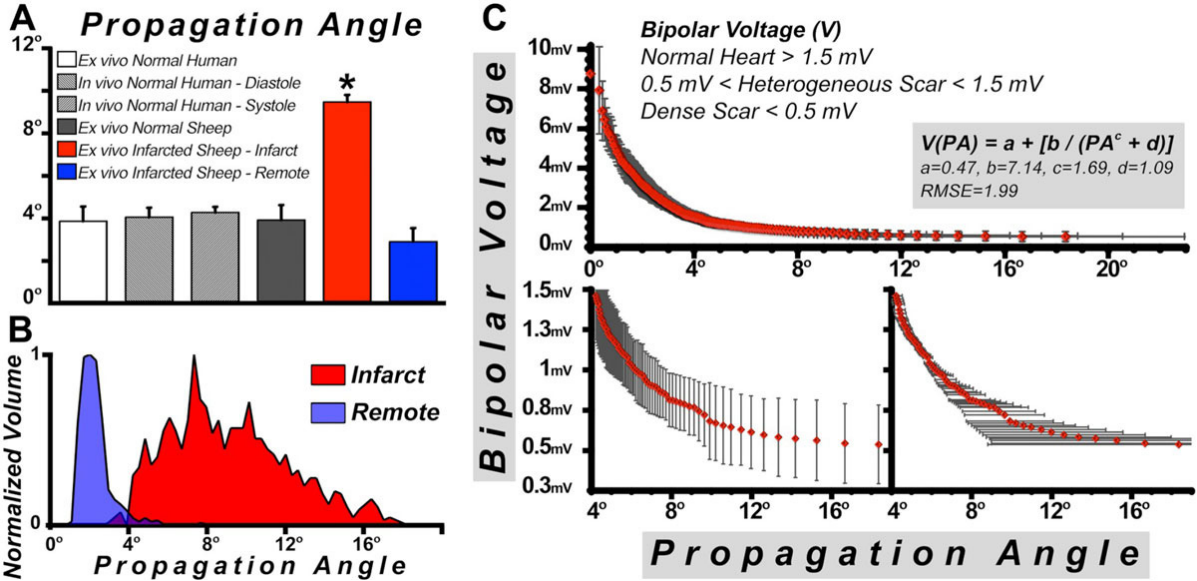
**(A) PA is approximately 4° in normal myocardium and is highly conserved across species**. Regions of infarction, however, show a significant increase (p < 0.05) in PA consistent with myofiber disorganization. (B) Infarcted myocardium is also characterized by a wide dispersion of PA. (C) Correlation between myocardial voltage and PA was determined by a rational polynomial curve. Regions of normal myocardium are characterized by voltage > 1.5 mV and PA < 4°. Regions with heterogenous scar have a voltage between 0.5-1.5 mV and PA between 4-10°. Dense scar is characterized by PA > 10° and a very low voltage.

Little variation in PA was observed between normal ovine and human myocardium **(**Figure 1A, B**)**. Marked differences, however, were seen in PA and bipolar voltages in the infarcted sheep **(**Figure 1C, D**)**. PA in normal sheep hearts was generally 2-4° **(**Figure 2A**) **but significantly higher in the infarcted hearts **(**Figure 2B**)**. Bipolar voltage (V) was used to segment the infarcted hearts into normal regions (> 1.5 mV), heterogeneous scar (0.5-1.5 mV), and dense scar (< 0.5 mV). PA was < 4° in electrically-normal myocardium, between 4° and 10° in heterogeneous scar, and > 10° in regions of dense scar **(**Figure 2C**)**. The relationship between V and PA was non-linear and could be approximated by the rational polynomial function V(PA) = a + [b/(PA^c ^+ d)], where a = 0.47, b = 7.14, c = 1.69, and d = 1.09 with root mean square error = 1.99.

## Conclusions

A narrow range of PA exists in normal hearts, and PA can be used to delineate regions of normal myocardium, heterogeneous scar, and dense scar. Regions of myocardium with very high PA values (> 10°) likely lack the structural coherence to support electrical conduction. PA values between 4-10°, however, correlate well with the bipolar voltage values seen in heterogeneous and arrhythmogenic scars. Recently, tractography of the entire human heart *in vivo *has become possible, which could allow PA to serve as a valuable tool in the assessment and management of patients at risk of sudden cardiac death.

## Funding

R01 HL093038 (D.E.S.), R01HL112831 (D.E.S.), P41RR14075 (Martinos Center).
